# Anti-colchicine Fab fragments prevent lethal colchicine toxicity in a porcine model: a pharmacokinetic and clinical study

**DOI:** 10.1080/15563650.2017.1422510

**Published:** 2018-01-15

**Authors:** Michael Eddleston, Nicolas Fabresse, Adrian Thompson, Ibrahim Al Abdulla, Rachael Gregson, Tim King, Alain Astier, Frederic J. Baud, R Eddie Clutton, Jean-Claude Alvarez

**Affiliations:** a Pharmacology, Toxicology, and Therapeutics, University/BHF Centre for Cardiovascular Science, University of Edinburgh, Edinburgh, UK;; b Wellcome Trust Critical Care for Large Animals, Royal (Dick) School of Veterinary Studies and the Roslin Institute, University of Edinburgh, Edinburgh, UK;; c Laboratoire de Pharmacologie – Toxicologie, Centre Hospitalier Universitaire Raymond Poincaré, AP-HP et MassSpecLab, Plateforme de Spectrométrie de Masse, Inserm U-1173, UFR des Sciences de la Santé Simone Veil, Université Versailles Saint-Quentin, Garches, France;; d Micropharm Ltd, Newcastle Emlyn, UK;; e School of Medicine Paris 12, Paris, France;; f University Paris Diderot, Assistance Publique - Hopitaux de Paris, Paris, France

**Keywords:** Colchicine, fab fragments, antidote

## Abstract

**Background:** Colchicine poisoning is commonly lethal. Colchicine-specific Fab fragments increase rat urinary colchicine clearance and have been associated with a good outcome in one patient. We aimed to develop a porcine model of colchicine toxicity to study the pharmacokinetics and efficacy of ovine Fab.

**Methods:** A Göttingen minipig critical care model was established and serial blood samples taken for colchicine and Fab pharmacokinetics, clinical chemistry, and haematology. Animals were euthanised when the mean arterial pressure fell below 45 mmHg without response to vasopressor, or at study completion.

**Results:** Initial studies indicated that oral dosing produced variable pharmacokinetics and time-to-euthanasia. By contrast, intravenous infusion of 0.25 mg/kg colchicine over 1 h produced reproducible pharmacokinetics (AUC_0–20_ 343 [SD = 21] µg/L/h), acute multi-organ injury, and cardiotoxicity requiring euthanasia a mean of 22.5 (SD = 3.2) h after dosing. A full-neutralising equimolar Fab dose given 6 h after the infusion (50% first hour, 50% next 6 h [to reduce renal-loss of unbound Fab]) produced a 7.35-fold increase in plasma colchicine (AUC_0–20_ 2,522 [SD = 14] µg/L/h), and removed all free plasma colchicine, but did not prevent toxicity (euthanasia at 29.1 [SD = 3.4] h). Earlier administration over 1 h of the full-neutralising dose, 1 or 3 h after the colchicine, produced a 12.9-fold (AUC_0–20_ 4,433 [SD = 607] µg/L/h) and 6.0-fold (AUC_0–20_ 2,047 [SD = 51] µg/L/h) increase in plasma colchicine, respectively, absence of free plasma colchicine until 20 h, and survival to study end without marked cardiotoxicity.

**Conclusions:** Colchicine-specific Fab given early, in equimolar dose, bound colchicine, eliciting its movement into the blood, and preventing severe toxicity. Clinical studies are now needed to determine how soon this antidote must be given to work in human poisoning.

## Introduction

Colchicine is used for the treatment of gout, familial Mediterranean fever, pericarditis, Behçet’s disease and increasingly for ischaemic heart disease [1–4]. It inhibits microtubule polymerization by binding to tubulin, preventing mitosis, and inhibiting neutrophil function. It has a narrow therapeutic index, resulting in severe toxicity after overdose, especially after intravenous (IV) administration or intentional poisoning [5–9]. Poisoning results in initial gastrointestinal effects that last for around 24 h and may be followed by multiorgan dysfunction or death from cardiogenic shock, usually within 48–96 h [8].

There is no licensed therapy, with current care revolving around supportive intensive care. Fab fragments [10] against colchicine were raised experimentally in the 1980–90s in France [10,11] and used on a compassionate basis in at least one patient [12]. They are now being produced commercially in sheep and have been tested in a rodent model, showing increased elimination of colchicine [13]. Ovine colchicine Fab fragments have not yet been studied in humans. Before this can happen, a pre-clinical model of colchicine poisoning similar to human poisoning is needed in which anti-colchicine Fab can be tested, to explore efficacious dosing and timing.

Pigs have been identified as good models for toxicology studies, offering similarities with human toxicity [14–16]. However, no pig model has been established for colchicine poisoning and the toxic dose is not known. Reviewing colchicine toxicity in humans, Harris and Gillett concluded that survival was typical with doses of <0.5 mg/kg while death was almost inevitable with doses >0.8 mg/kg, although acknowledging multiple reports of fatalities after exposure to <0.5 mg/kg [6]. Rats probably do not offer an appropriate model for human toxicity because they are highly resistant to colchicine poisoning with an LD50 > 25 mg/kg following oral dosing [17].

We therefore established a model of acute colchicine poisoning in Gottingen minipigs, initially testing oral then IV administration. This model showed that early administration of colchicine Fab produced effective redistribution of colchicine from tissues into the vascular space and prevented lethal colchicine toxicity. A clinical study is now required in human patients to assess the appropriate dose and maximum interval to effective therapy.

## Methods

### Animals and ethics

Experiments involved male Göttingen minipigs (Ellegaard Minipigs ApS, Dalmose, DK) with mean body mass 30.4 (SD 3.1) kg, *n* = 14. Animals were barrier bred, shown to be free of infection before shipment, and treated in accordance with the Animals (Scientific Procedures) Act of 1986. This study was performed under Home Office Licence after institutional ethics review.

### Colchicine and anti-colchicine fab fragments

Colchicine (Abcam, Cambridge, UK) solutions were made up in sterile water for injection at 1.0 mg/mL and filter sterilised using a 0.2-µm in-line filter. Colchicine containers were wrapped in metal foil and stored in the dark to prevent photodegradation. Solutions were diluted to 0.5 and 0.0625 mg/mL for oral and IV administration, respectively, and injected volumes adjusted to give the required dose (mL/kg) based on the animal’s body mass on the study day. The dose varied from 0.25 to 1.0 mg/kg. Oral doses were given via nasogastric tube by bolus push using a 60-mL catheter tip syringe; IV doses were infused over 1 h.

Anti-colchicine Fab (ColchiBIND) was supplied as a solution in 10 mL ampoules by Micropharm Ltd, with each containing sufficient Fab to neutralise approximately 0.2 mg colchicine. The average affinity constant (Ka) of the ovine Fab was 3.16 × 10^11^ M^−1^ (Micropharm Ltd, Unpublished). The Fab volume administered was varied according to the pig’s body mass on the study day. The ColchiBIND solution was drawn up into 50 mL syringes fitted with a 0.2-µm in-line filter immediately before infusion.

### Study design

Drug-naive animals were kept in pens and acclimatised under the care of institutional veterinary surgeons before the study. Animals were fed a standard diet but fasted overnight preceding the study; water was available freely. Animals were weighed on the morning of the study.

The experiment was conducted in two phases, with the first phase being used to identify a toxic lethal dose with survival lasting greater than 24 h and death from apparent cardiotoxicity. Based on the proposed human lethal dose of colchicine being between 0.5 and 1.0 mg/kg, we used two single doses of oral colchicine (0.5 or 1.0 mg/kg). The second phase was used to study the pharmacokinetic (PK) and clinical effects of anti-colchicine Fab on lethal colchicine toxicity, using 1 of 2 single doses of intravenous colchicine (0.25 or 1.0 mg/kg). We included the lower dose in case intravenous colchicine was more toxic than orally administered colchicine. Once the model was established, pharmacokinetic (PK) and clinical effects of therapeutic administration of ovine colchicine Fab fragments were evaluated at various times and infusion durations after administration of a toxic intravenous dose of colchicine.

To reduce bias, animals were allocated to treatment groups using a random number list; allocation could not be predicted before randomization. No data were available for calculating group sizes in this model; however, the use of anti-colchicine Fab at different times post poisoning offered a temporal basis for testing clinical efficacy as well as assessing the PK.

### Anaesthesia and instrumentation

Studies began each day at around 07:30. Studies using oral administration were planned to last up to 60 h; after the switch to IV colchicine, the studies lasted for up to 48 h. The methods of anaesthesia induction, central vascular access, and instrumentation together with continual cardiovascular and respiratory monitoring of animals have been described previously [18].

### Experimental protocol

After a 30-min stabilization period for the pigs after instrumentation, colchicine solution was given by oral bolus dose via NG tube (0.5 and 1.0 mg/kg) or by IV infusion over 1 h via a central venous line (0.25 or 1.0 mg/kg. The volume of colchicine solution varied according to the pig’s body mass. Animals received an infusion of 5 mL/kg/h 0.9% sodium chloride during the study, increased as clinically required. Norepinephrine (4 mg/100 mL solution) was given IV if the mean arterial pressure (MAP) fell below 45 mmHg and did not respond to fluid resuscitation.

In the Fab fragment studies, to fully neutralise the 0.25 mg/kg colchicine challenge, 12.5 mL/kg (e.g. 375 mL for a 30 kg animal) of Fab fragments were administered. The single clinical case in the literature reported cardiovascular improvement and impressive PK response (marked redistribution of colchicine into the blood) to Fab given 40 h after exposure [12]. Being conservative, but wishing to select a clinically relevant time point for administration, we first chose to give Fab 7 h after the start (and 6 h after the end) of the colchicine infusion. The dose was split into two, with one half given over 1 h followed by the second half over 6 h, to reduce wastage in the urine of free Fab, unbound to colchicine (as done for digoxin specific Fab) [19,20]. Since this proved ineffective, in further studies, to increase the likelihood of success, the full neutralising dose was given earlier and administered over 1 h.

***Sampling***. Arterial blood samples were taken for pharmacokinetic analysis at −30 and −10 min prior to colchicine, at 5, 10, 15, 30, 45, 60 and 90 min post dosing, and then at 2, 4, and 4 h intervals until 24 h, and 8 hourly thereafter until the animal’s euthanasia. The sample was transferred to an EDTA-containing tube, centrifuged for 7 min at 3900 rpm, and the plasma stored frozen at −20 °C until analysis.

Arterial blood gasses and clinical chemistry were monitored on an EPOC blood analyser (Woodley Equipment Company Ltd, UK) pre-treatment and at 30, 60 and 90 min, 2 and 4-h post-dosing, and at 4-h intervals thereafter. Blood chemistry was checked pre-treatment and at 8-h intervals post treatment; hematology including prothrombin times was checked pre-treatment and at 12-h intervals thereafter. Blood samples were taken pre-treatment, after 24 h, and immediately before euthanasia, for microbiology studies).

Urine was collected at 3 h intervals throughout the study through an indwelling bladder catheter. Urine from the previous three hours was mixed and two 10 mL samples taken off and immediately frozen.

Samples of heart (left ventricle), muscle (*tibialis cranialis*), liver, lung, and small intestine were taken post-mortem and stored in formal saline before histological analysis.

***Euthanasia***. Animals were given an overdose of IV sodium pentobarbital whilst under isoflurane anaesthesia either at the end of the planned study (48 h) or when their MAP fell below 45 mmHg for 30 min and continued to fall without sign of recovery despite fluids and vasopressors.

### Statistical analysis

Formal power calculations could not be performed due to a lack of data for the effect of colchicine in pigs. Primary data analysis was done in Prism 7.0 (GraphPad, CA). All animals were included in the analysis. Pig weights, clinical and biochemical outcomes were summarized with mean and SD. Time to euthanasia was assumed not to be normally distributed and hence is described with median and interquartile range (IQR), while acknowledging the small sample size. Individual animal data are provided to explicitly illustrate time to euthanasia.

### Measurement of colchicine concentrations

Free and Fab-bound colchicine was measured using a LC-MS/MS method, as published [21]. Briefly, free colchicine was separated from Fab-bound colchicine using 30 KDa microfilters. Total colchicine was determined after Fab denaturation by dilution in water and heating at 100 °C for 1 h. Deuterated colchicine was used as internal standard. Samples were then processed by liquid-liquid extraction before LC-MS/MS analysis. Calibration curves were designed over the concentration range 0.5–100 ng/mL for plasma and urine.

### Measurement of anti-colchicine fab fragment concentrations

An ELISA was developed to measure Fab concentrations in plasma and urine. Calibration curves (0.01–0.5 mg/L) were generated using sheep IgG Fab fragments (Rockland Immunochemicals) as standards. Accuracy and precision were controlled using three levels of quality control (QC) samples (0.03, 0.08 and 0.125 mg/L). Standards and QC were prepared in porcine plasma. Well plates were coated with 2 mg/L of rabbit anti-sheep IgG F(ab’)_2_ fragments (Jackson ImmunoResearch, West Grove). Fab standards and samples were added on corresponding wells. After 1 h incubation at 37 °C, peroxidase-conjugated rabbit anti-sheep IgG F(ab’)_2_ was added and plates incubated for 30 min at 37 °C. Plates were washed, 3,3′,5,5′-tetramethylbenzidine added, incubated for 15 min, and a stop solution (HCl 1M) added. Absorbance was measured using a POLARstar omega plate reader (BMG Labtech, Ortenberg, Germany) at 450 nm.

### Pharmacokinetic analysis

Plasma colchicine and Fab concentration time data were analysed using the MicroPharm Kinetics 5.0 software, using a two-compartment models. The log linear concentration time data were fitted by linear regression analysis to obtain the volume of distribution; terminal disposition constants (α and β) were calculated by mean square regression analysis. Distribution and terminal half-lives (respectively, *t*_1/2α_ and *t*_1/2β_) were calculated as ln2/α and ln2/β. AUC_0–20h_ and AUC_0–end_ were calculated by use of the linear trapezoidal rule. AUC_0–∞_ was calculated for Fab by adding to AUC_0–48_ the value of the last measured plasma concentration divided by terminal disposition rate constant. QU_0–20_ and QU_0–48_ are the cumulated amounts of unchanged colchicine excreted in the urine from the time 0–20 and 0–48 h, respectively.

### Funding

The funders had no role in study design, data collection, data interpretation, or writing of the report. Micropharm staff performed the measurement of plasma Fab concentrations but the PK analysis was done by others. The authors had full access to all the study data; all agreed with the decision to submit for publication. The first author (ME) guarantees the data within the paper.

## Results

### Oral colchicine administration

**Figure 1.**
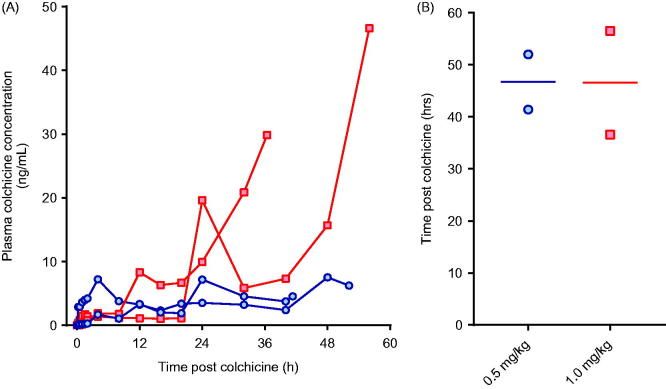
(A) Plasma colchicine concentration and (B) time to euthanasia for animals receiving 0.5 mg/kg (blue circles) or 1.0 mg/kg (red squares) colchicine by oral administration.

We first attempted to identify an oral dose of colchicine that would cause severe toxicity, matching human poisoning after ingestion of colchicine tablets. In a dose-ranging study, four pigs were randomly administered 0.5 or 1.0 mg/kg of colchicine in solution by oral gavage and studied for 60 h. This did not result in a reproducible model, with variable PK profiles for plasma colchicine concentration and no dose–response in the time-to-euthanasia (Figure 1).

**Figure 2.**
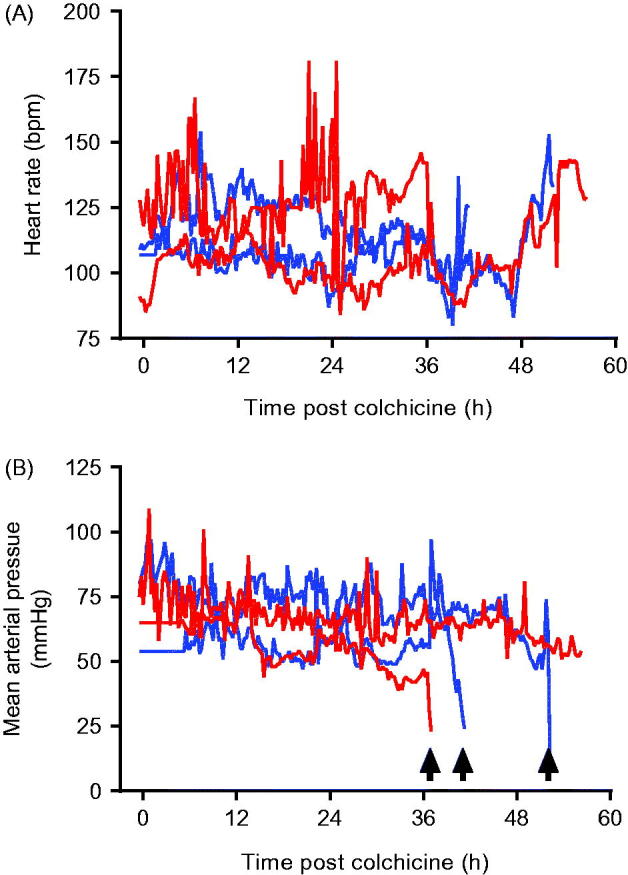
(A) Heart rate and (B) mean arterial pressure in animals receiving 0.5 mg/kg (blue lines) or 1.0 mg/kg (red lines) colchicine by oral administration. Arrows mark sudden catastrophic cardiovascular collapse in three of the animals.

The animals did, however, show evidence of multi-organ injury and severe cardiotoxicity. Cardio-respiratory function was not affected by colchicine until late in the poisoning (Figure 2). In three of four pigs (two 0.5 mg/kg, one 1.0 mg/kg), cardiotoxicity developed with a fall in MAP requiring norepinephrine. The doses of norepinephrine required to maintain an MAP > 45 mmHg increased rapidly over a few hours before both heart rate and MAP fell precipitously over minutes (Figure 2) and the animals were euthanised. The fourth (2nd high dose) animal began to develop cardiotoxicity after 53 h; norepinephrine requirements rose more slowly than with the other animals. It was euthanised as severe cardiotoxicity developed at 56.5 h.

Clinical biochemistry analyses showed evidence of liver (raised AST activity) and muscle (raised CK activity) injury after 15 h as well as late renal injury in the hours before euthanasia (data not shown).

### Intravenous colchicine administration

To seek a more reliable model, we switched to the IV route of administration for the next four pigs (a route previously used for clinical therapy [7]). To compensate for increased colchicine bioavailability after IV administration [8], the lower dose was halved to 0.25 mg/kg while a higher dose was kept at 1.0 mg/kg with the ambition of bracketing severe toxicity. The colchicine was infused over one hour.

The first two pigs received 0.25 and 1.0 mg/kg by IV infusion. Because toxicity was rapid for the higher dose, with euthanasia required at 14.5 h, the next two animals received 0.25 mg/kg IV.

**Figure 3.**
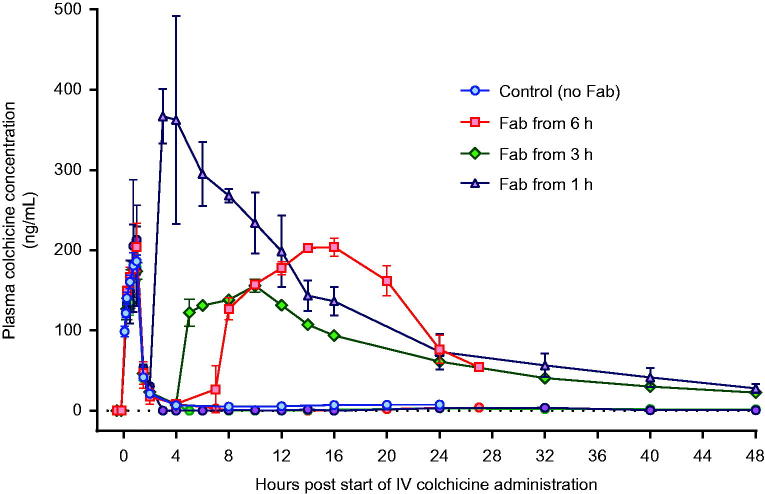
Plasma pharmacokinetics for total colchicine and free colchicine in animals receiving intravenous colchicine (0.25 mg/kg), with or without anti-colchicine Fab, according to delay to treatment. Control animals received no Fab after IV colchicine 0.25 mg/kg over 1 h = blue squares. Animals received Fab at 6 h post-infusion (total: red square; free colchicine: red circle), Fab at 3 h post-infusion (total: green diamond; free colchicine: green circle), and Fab at 1 h post-infusion (total: purple triangle; free colchicine: purple circle).

Colchicine PK was similar in the three animals receiving 0.25 mg/kg, with a mean AUC_0–20_ of 343 µg/L/h (SD = 21 µg/L/h), *t*_1/2α_ of 0.21 h (SD = 0.012 h) and QU_0–20_ of 17% (SD = 2.9%) (Figure 3; Table 1). In the animal receiving the higher dose, AUC_0–16_ was 2,398 µg/L/h and *t*_1/2α_ 0.15 h. The elimination half-life could not be calculated because of a plateau in plasma colchicine concentration from 8 h post-administration to time of euthanasia.

**Table 1.** Pharmacokinetics of colchicine and Fab.

	Colchicine administered IV 0.25 mg/kg						
Fab infusion	Controls	From 1 h after the colchicine infusion		From 3 h after the colchicine infusion		From 6 h after the colchicine infusion	
Animals	*n* = 3 (mean ± SD)	*n* = 1	*n* = 1	*n* = 1	*n* = 1	*n* = 1	*n* = 1
AUC_0–20H_ (µg L^−1^ h^−1^)	343 ± 21	4862	4004	2011	2083	2532	2512
Free colchicines AUC_0–20_ (µg L^−1^ h^−1^)		389	259	298	285	300	220
AUC_0–end_ (µg L^−1^ h^−1^)		3741	5313	3142	3343	3182	2971
Ratio (AUC_0–20_/AUC_0–20_ control)	1	14.1	11.6	5.8	6.1	7.4	7.3
*t*_1/2α_ (h)	0.21 ± 0.12	*—*	*—*	*—*	*—*	0.21	0.19
*t*_1/2β_ (h)	*—*	9.7	9.0	11	11	*—*	*—*
QU_0–20h_	17 ± 2.9%	38%	47%	37%	36%	18%	18%
QU_0–end_		43%	56%	43%	43%	18%	20%
Ratio (QU_0–20_/QU_0–20_ control)	1	2.2	2.8	2.2	2.1	1.1	1.1
Fab AUC_0-∞_ (g L^−1^ h^−1^)	*—*	40.0	32.4	28.2	31.9	*—*	*—*
Fab *t*_1/2α_ (h)	*—*	2.3	2.6	1.6	1.3	*—*	*—*
Fab *t*_1/2β_ (h)	*—*	44.4	31.3	22.1	11.5	*—*	*—*

The Fab infusions given 1 and 3 h after the colchicine infusion were administered over 1 h; the Fab infusion given 6 h after the colchicine infusion finished was administered over 7 h (50% over first hour, 50% over following 6 h).

**Figure 4.**
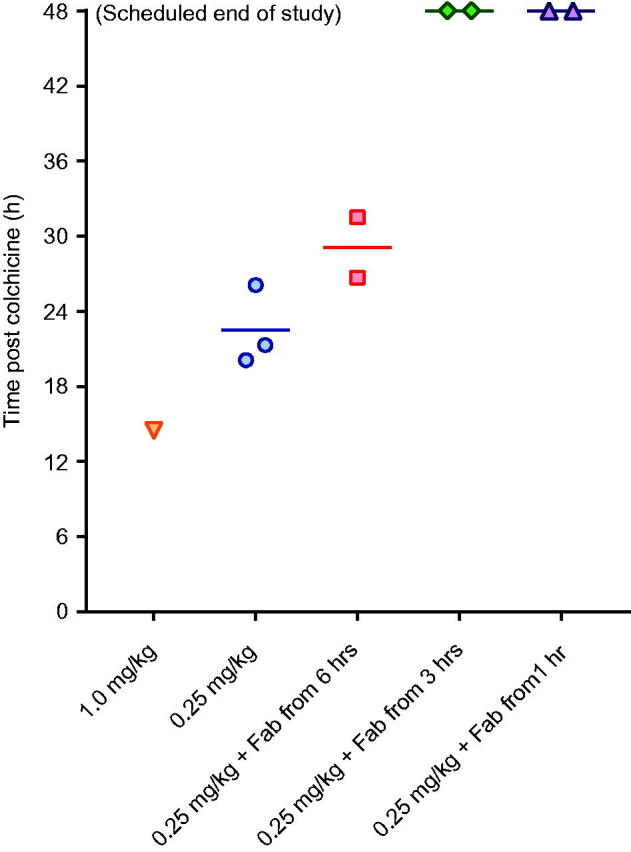
Time to euthanasia in animals receiving intravenous colchicine with or without anti-colchicine Fab, according to delay to treatment.

**Figure 5.**
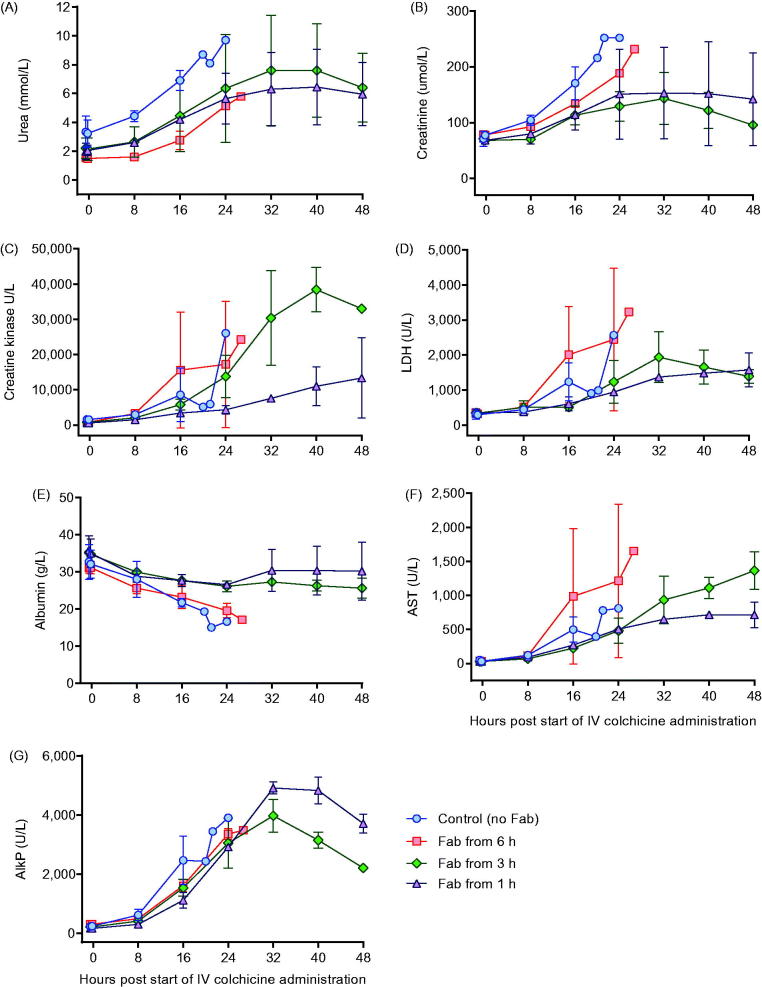
Clinical biochemistry in animals receiving intravenous colchicine (0.25 mg/kg), with or without anti-colchicine Fab. Control animals received no Fab after IV colchicine 0.25 mg/kg over 1 h (blue circles). Animals received Fab at 6 h post-infusion (red square), Fab at 3 h post-infusion (green diamond), and Fab at 1 h post infusion (purple triangle). (A) urea, (B) creatinine, (C) creatine kinase, (D) lactate dehydrogenase (LDH), (E) albumin, (F) aspartate aminotransferase (ALT) and (G) alkaline phosphatase (AlkP).

**Figure 6.**
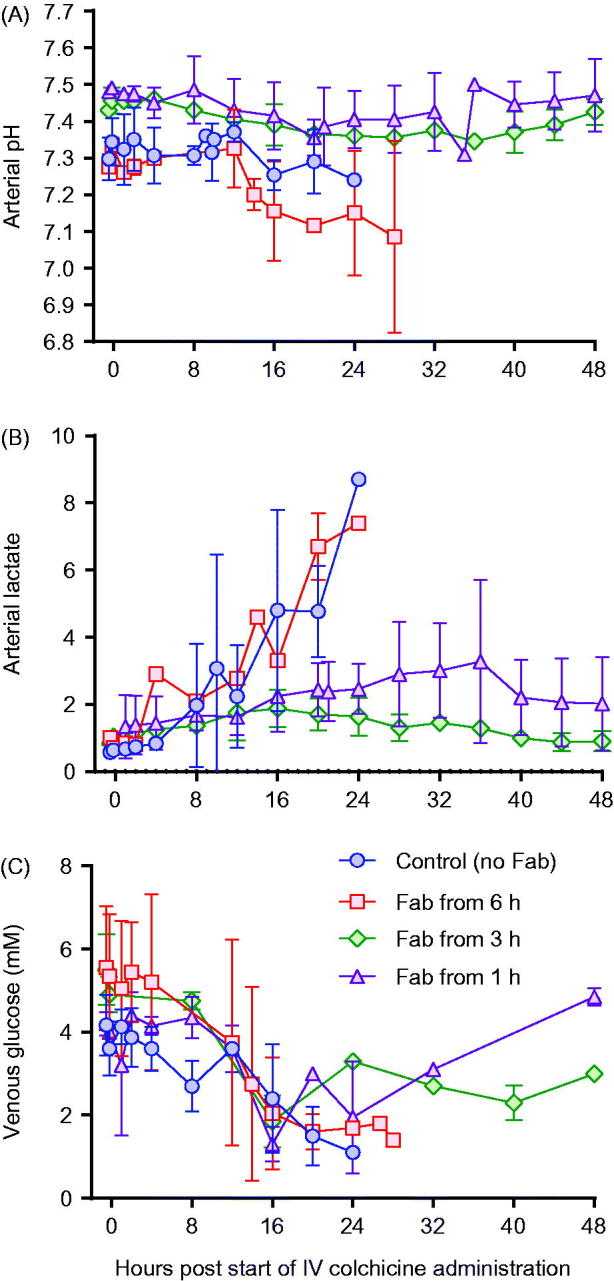
Blood gas analysis in animals receiving intravenous colchicine (0.25 mg/kg), with or without anti-colchicine Fab. Control animals received no Fab after IV colchicine 0.25 mg/kg over 1 h (blue circles). Animals received Fab at 6 h post-infusion (red square), Fab at 3 h post-infusion (green diamond), and Fab at 1 h post-infusion (purple triangle). (A) arterial pH, (B) arterial lactate concentration and (C) venous glucose concentration.

Toxicity was consistent in the three animals receiving 0.25 mg/kg, with euthanasia required a mean of 22.5 (SD 3.2) h after starting the infusion (Figure 4). Animals showed evidence of dose-related liver, renal and muscle toxicity (Figure 5), as well as late, severe cardiotoxicity. Blood gas analysis showed no effect on arterial pH but a substantial rise in arterial lactate concentration, peaking at 7–9 mmol/L (Figure 6). No bacteria were cultured from samples taken for microbiology. Histology showed moderate, diffuse necrotizing enteritis of the small intestine, mild cardiac myopathy, and pulmonary alveolar and interlobular oedema.

The IV dose of 0.25 mg/kg colchicine was selected to study the effect of anti-colchicine Fab.

### Administration of anti-colchicine fab to colchicine poisoned pigs

A full neutralising dose of anti-colchicine Fab given from 6 h after completion of the colchicine infusion (50% over 1 h, 50% of the next 6 h) produced a 7.35-fold increase in plasma colchicine (AUC_0–20_ 2,522 [SD = 14] µg/L/h) and removed all free colchicine during the infusion (Figure 4). However, it did not increase urinary elimination of colchicine (QU_0–20_ 18%, mean of 1.1-fold greater than controls) and did not prevent toxicity or markedly improve outcome—euthanasia was required a mean of 29.1 (SD 3.4) h after starting the colchicine.

Infusion over 1 h of a full neutralising dose of anti-colchicine Fab, 1 and 3 h after the end (2 and 4 h after the start) of the colchicine infusion, markedly increased both plasma colchicine and urinary elimination of colchicine. The Fab produced 12.9-fold (mean AUC_0–20_ 4,433 [SD = 607] µg/L/h) and 6.0-fold (AUC_0–20_ 2,047 [SD = 51] µg/L/h) increases in plasma colchicine, as well as 2.5-fold (QU_0–20_ 42.5 [SD = 6.4] %) and 2.1-fold (AUC_0–20_ 36.5 [SD = 0.7] %) increases in urinary colchicine elimination, respectively.

This increased extraction of colchicine from the tissues and elimination was associated with markedly reduced toxicity, with all four animals surviving to 48 h (Figure 4) and none showing marked cardiotoxicity and cardiovascular decompensation (as seen for animals not receiving Fab—see Figure 2) (data not shown). Clinical biochemistry showed reduced liver, kidney and muscle injury (Figure 5). Blood gas analysis showed no effect on arterial pH or lactate concentration, except for transient rise in lactate to 5 mmol/L in one animal receiving Fab 1 h after the end of the colchicine infusion (Figure 6). No bacteria were cultured from samples taken for microbiology. Histology showed mild necrotizing enteritis of the small intestine, mild cardiac myopathy, and mild pulmonary alveolar and interlobular oedema.

The Fab had a mean *t*_1/2α_ of 2.0 (SD 0.6) h and mean *t*_1/2β_ of 27.3 (SD 14.0) h (Table 1). The half-life of elimination was slow enough in all animals to keep concentrations of free plasma colchicine undetectable until 20 h and at very low concentrations until the end of each study.

## Discussion

This study has shown that early administration of anti-colchicine Fab fragments redistributes colchicine from extracellular fluid to the blood and increases its elimination in the urine; this removal of colchicine from the tissues, if the Fab is given sufficiently early, in sufficient dose, prevents colchicine-induced cardiotoxicity, multi-organ injury, and metabolic acidosis. Although a small translational study, the results suggest that anti-colchicine Fab may benefit patients with colchicine toxicity if given early enough after exposure.

Administration of a full neutralising dose of Fab within 3 h of the end of colchicine administration resulted in marked increases in plasma colchicine and urinary elimination, and absence of free colchicine from plasma until 20 h after colchicine exposure. Delay of Fab administration to 6 h after the end of colchicine administration, and administration of a half-neutralising dose, resulted in effective redistribution—similar to that following Fab started at 3 h—but a less clear effect on urinary elimination and only a modest increase in time to euthanasia. Although this poor delayed effect may be due to the Fab dose administered, our results from IV dosing may suggest that colchicine Fab will be clinically relevant for relatively few patients since many present to hospital after 6 h [5,6].

This possible lack of effectiveness after only a few hours contrasts with the single report of a patient treated with goat anti-colchicine antibodies which showed both PK and clinical benefit when receiving Fab 40 h after ingestion [12]. This difference might be because porcine colchicine poisoning is not a perfect model of human poisoning, with differences between pigs and humans in cardiovascular function and metabolism. However, we found the lethal dose in pigs to be similar to that reported for humans, and far lower than the rat LD50 (>25 mg/kg), consistent with some relevance of pig poisoning for modelling human poisoning. Multi-organ injury seen in the model also replicated human poisoning. The polyclonal Fab had very high affinity for colchicine (average affinity constant 3.16 × 10^11^ M^−1^) suggesting that differences in affinity between the goat antibody used for the clinical case (affinity 2.0 × 10^10^ M^−1^) and the ovine Fab used here is not responsible for the differences in effect. It is also possible that the pharmacokinetics of the ovine Fab in this pig model are quite different to that of the goat Fab used in the clinical case [12].

Unfortunately, oral colchicine dosing did not produce reliable PK or model poisoning well, likely due to variation in absorption of the colchicine from the anaesthetised pig gut. This variability was not noted in our previous studies of oral dimethoate organophosphorus insecticide toxicity [18], probably due to the lower toxicity of the insecticide and therefore much higher doses needed for poisoning (1000 mg/kg).

This pig study suggests that humans will have to be treated soon after poisoning for the Fab to be effective. This might be true for very high doses but not for more borderline doses. Increasing the number of animals studied with IV colchicine at different delays to Fab is unlikely to yield further information on timing and doses for colchicine tablet poisoning. Intravenous infusions over 1 h may not accurately reflect oral poisoning despite similar *C*max and bioavailability. The slower absorption and distribution to tissues, and direct effect on gut enterocytes, of oral administration may allow the Fab longer to work in human poisoning [22].

Instead, a clinical study is needed that treats all patients presenting to hospital with colchicine poisoning up to 48 h after exposure, stratified according to time to treatment, estimated dose ingested, and plasma colchicine concentration before treatment. Due to the scarcity of cases, the study will need to be observational. Such a study is now being started in France, based on the data on redistribution reported in this porcine study. As patients are studied, and outcomes recorded, the time to treatment could be slowly shortened down to 6, 12 or 24 h. Alternatively, all patients could in future be treated with the understanding that only borderline cases will get any benefit after a certain time.

The pharmacokinetics of colchicine and Fab in the pigs were generally similar to those reported in humans. The distribution half-life of colchicine (0.21 h) and of Fab (2.0 h) in this porcine study were similar to that observed in humans (colchicine 0.15 h, anti-digoxin Fab 0.5–1.9 h) [21,23]. Distribution was complete one hour after the end of infusion, consistent with the best response to Fab occuring when it was started at 1 h. Fab administration increased the AUC 6 to 14-fold compared to controls, similar to that noted in colchicine intoxicated rabbits (19-fold) [24]. The elimination half-lives (9.0–11.0 h) calculated in the last four pigs were shorter than observed in humans receiving IV colchicine infusions (30 ± 6 h). In the same way, elimination half-life (11.5–44.4 h) was comparable to half-life in human (11.0–34.5 h) exhibiting a high variability.

### Limitations

We used a PK dose–response approach to minimise the number of animals used in the study before moving into human studies. Despite being a small study, robust dose/time-response effects were seen in terms of the AUC, time to euthanasia, and reduction in organ injury. Since the study has provided proof of binding *in vivo* in a clinically relevant model that is now leading to clinical trials, use of further animals is unlikely to provide additional useful data. We acknowledge that there are cardiovascular and metabolic differences between pigs and humans—such as basal heart rate and mass of the liver and kidneys—that might make pigs more sensitive to cardiotoxicity, reducing effectiveness compared to humans. However, additional pig studies will not help guide human treatment.

We used IV infusions of colchicine over 1 h to simulate oral dosing due to the unreliable PK of oral colchicine in the anaesthetised pigs. This difference might be important although the bioavailability of oral tablets is similar to IV infusions [5,6]. The animals receiving Fab at 6 h received only half a neutralising dose over the first hour, followed by the second half dose over 6 h. It is therefore not possible to directly compare the results with the 1 and 3 h delays to administration. However, redistribution was similar to that after a 3 h delay, despite the lack of effect on severity. This information is useful and reinforces the need for a large clinical study to identify the latest possible treatment time in human patients. The IV dose of 0.25 mg/kg was selected as an apparently reliable lethal dose of colchicine; we did not explore further doses between 0.25 and 1.0 mg since our aim was to rapidly move into human studies once we had tested whether the Fab could bind and redistribute colchicine with clinical effect *in vivo* in the pig.

## Conclusion

In this clinically relevant porcine model of colchicine poisoning, anti-colchicine Fab fragments were highly effective at stopping toxicity if given early enough in a large enough dose. Clinical trials are required to translate this knowledge into human patients.
